# Soybean Root Growth in Response to Chemical, Physical, and Biological Soil Variations

**DOI:** 10.3389/fpls.2021.602569

**Published:** 2021-02-23

**Authors:** Mariele Müller, Julia Renata Schneider, Vilson Antônio Klein, Eliardo da Silva, José Pereira da Silva Júnior, Adriano Mendonça Souza, Geraldo Chavarria

**Affiliations:** ^1^Agronomy Post-Graduate Program, Faculty of Agronomy and Veterinary Medicine, University of Passo Fundo, Passo Fundo, Brazil; ^2^Faculty of Agronomy and Veterinary Medicine, University of Passo Fundo, Passo Fundo, Brazil; ^3^Embrapa Wheat, Passo Fundo, Brazil; ^4^Department of Statistics, Federal University of Santa Maria, Santa Maria, Brazil

**Keywords:** *Glycine max* (L.) Merril, root volume, soil nutrition, soil porosity, principal component analyses

## Abstract

Environmental conditions affect crop yield, and water deficit has been highlighted by the negative impact on soybean grain production. Radicial growth in greater volume and depth can be an alternative to minimize losses caused by a lack of water. Therefore, knowledge of how soybean roots behave before the chemical, physical, and biological attributes of the soil can help establish managements that benefit in-depth root growth. The objective was to evaluate the growth of soybean roots in response to chemical, physical, and biological variations in the soil, in different soil locations and depths. Six experiments were conducted in different locations. Soil samples were collected every 5 cm of soil up to 60 cm of soil depth for chemical, physical, and biological analysis. The roots were collected every 5 cm deep up to 45 cm deep from the ground. The six sites presented unsatisfactory values of pH and organic matter, and presented phosphorus, potassium, and calcium at high concentrations in the first centimeters of soil depth. The total porosity of the soil was above 0.50 m^3^ m^−3^, but the proportion of the volume of macropores, micropores, and cryptopores resulted in soils with resistance to penetration to the roots. Microbial biomass was higher on the soil surface when compared to deeper soil layers, however, the metabolic quotient was higher in soil depth, showing that microorganisms in depth have low ability to incorporate carbon into microbial biomass. Root growth occurred in a greater proportion in the first centimeters of soil-depth, possibly because the soil attributes that favor the root growth is concentrated on the soil surface.

## Introduction

The productivity of soybean [*Glycine max* (L.) Merril] has shown a constant increase over the years, mainly due to the new technologies and management techniques used (Hesler et al., 2013; Hartman et al., 2015). Eventually, edaphoclimatic conditions negatively affect crop yields, and water stress has been shown to have the environmental condition that has the greatest negative impact on production (Fahad et al., 2017).

The sustainability of soybean yields is threatened by climate change, and water restriction events are becoming common in many parts of the world (Foyer et al., 2016). Given this imminent threat to protein food reduction, it is necessary to select soybean cultivars more drought tolerant (Foyer et al., 2016). Recent advances in understanding drought effects on soybean growth have been predominantly based on the evaluation of above-ground characteristics. In contrast, the impact of drought on soybean roots has been less studied (Kunert et al., 2016).

Water is absorbed by the roots, so providing adequate conditions for radicial growth, in volume and depth, can be an alternative to decrease the stress caused by the lack of water; thus, the roots will explore greater soil volume, consequently, will be in contact with a higher volume of water. The lack of water to the plants causes the closure of the stomata and decreases the plants' photosynthetic rate, causing losses in soybean grain production (Zhang et al., 2016; Kelly et al., 2019).

The different management adopted by farmers result in chemical, physical, and biological characteristics distinct from the soil, and may have reflexes in plants' root growth (Ahmed et al., 2019). Generally, the chemical restrictions occur due to the absence of essential elements and/or the presence of toxic elements; the physical restriction occurs mainly by increasing the soil density that increases the penetration resistance and can decrease the soil porosity, and the biological one by affecting the number of soil microorganisms that are beneficial to plants (Cardoso et al., 2013).

It is important to understand the phenotypic plasticity of roots in relation to the cultivation environment, due to the impact that this variable can present on plant production. So, evaluating the soybean root system can help to understand interactions with the cultivation environment, indicating the possible management practices aimed at increasing soil exploitation by roots and maintaining productive crop stability.

This research seeks to understand how soybean roots grow in soils with distinct chemical, physical, and biological attributes. The hypothesis is that soils with high quality in these attributes, benefit the roots in greater soil depth. The aim was to evaluate the soybean roots growth in response to chemical, physical, and biological soil variations in different sites and soil depths.

## Materials and Methods

### Site Description

The soybean cultivar used in the experiments was DM53i54 RSF IPRO. The experiments were conducted in 2017/2018 and 2018/2019, in three sites each year, totaling six sites: (i) **Site 1** and **Site 2**, municipality of Coxilha, Rio Grande do Sul state (RS), Brazil (28°07′ S; 52°17′ W; 721 m altitude) in 2017/2018; (ii) **Site 3**, municipality of Passo Fundo, RS, Brazil, (28°12′ S; 52°23′ W; 667 m altitude) in 2017/2018; (iii) **Site 4** and **Site 5**, municipality of Coxilha in 2018/2019; and (iv) **Site 6**, municipality of Passo Fundo in 2018/2019.

### Experimental Design

The six sites were conducted in different areas of cultivation, so they were not considered to be repetitions, but different sites, because even though the sites are close, the managements adopted in the areas are distinct and therefore resulted in differences in the chemical, physical and biological attributes of the soil. Sites 1, 2, 4, and 5 are areas managed with crop rotation, mainly with the species black oat (*Avena strigosa* Schreb.), wheat (*Triticum* spp.), sudangrass (*Sudanese sorghum*), soybean, and corn (*Zea mays*). In 2016, in Sites 1 and 4 the soil was subsoiling. Places 3 and 6 are areas managed with crop succession, being wheat in winter and soybean in summer.

All sites are in a humid subtropical climate region, with humic dystrophic Red Latosol soil (Oxisol) (Streck et al., 2008), and with the consolidated no-tillage system. These soils originated from basalt had higher clay content, in which the 1:1 type clay mineral (kaolinite) and iron oxides (hematite and goethite) predominate (Ajayi et al., 2009).

Sowing in the six sites was performed with stem furrow. Sowing at **Sites 1** and **2** was held on October 17th, 2017, at **Site 3** on November 3rd, 2017, at **Sites 4** and **5** on October 13th, 2018, and at **Site 6** on November 5th, 2018. At **Sites 1**, **2**, **4**, and **5** the sowing was performed with base fertilization of 15 kg ha^−1^ of N, 87 kg ha^−1^ of P_2_O_5_, and 21 kg ha^−1^ of K_2_O, before sowing 200 kg ha^−1^ of potassium chloride (KCl) was applied to the ground at launch. At **Site 3** and **6**, fertilization at sowing was 6 kg ha^−1^ of N, 60 kg ha^−1^ of P_2_O_5_, and 60 kg ha^−1^ of K_2_O.

At **Sites 3** and **6**, the plots were 10 m long and 10 rows were sowing, with five replications, the repetitions were side by side. At **Sites 1**, **2**, **4**, and **5** the plots were 15 m long and 10 rows of sowing with five replications, the repetitions were following the sowing lines. The cultivation areas did not present soil spots, and the collections always occurred in the same sowing line in each repetition.

Plant density in the six sites was 10.8 plants m^−1^, totaling 240,000 plants ha^−1^, with spacing between lines of 0.45 m. The phenological scale of Fehr and Caviness (1977) was used to determine the phenological stages of soybean.

The mean content of sand, clay, and silt of the six sites up to 60 cm of soil depth was determined (Table 1).

**Table 1:** Percentage of sand, clay, and silt in the six sites up to 60 cm in soil depth.

**Site**	**Sand (%)**	**Clay (%)**	**Silt (%)**
**Site 1**	11.17	66.25	22.58
**Site 2**	10.89	68.02	21.09
**Site 3**	29.74	54.87	15.39
**Site 4**	9.30	74.09	16.62
**Site 5**	12.25	66.83	20.92
**Site 6**	32.94	55.23	11.83

At the R5 phenological stage, five trenches for the site were made for sampling and soil collection for chemical, physical and biological analysis, and root growth.

**Figure 1:**
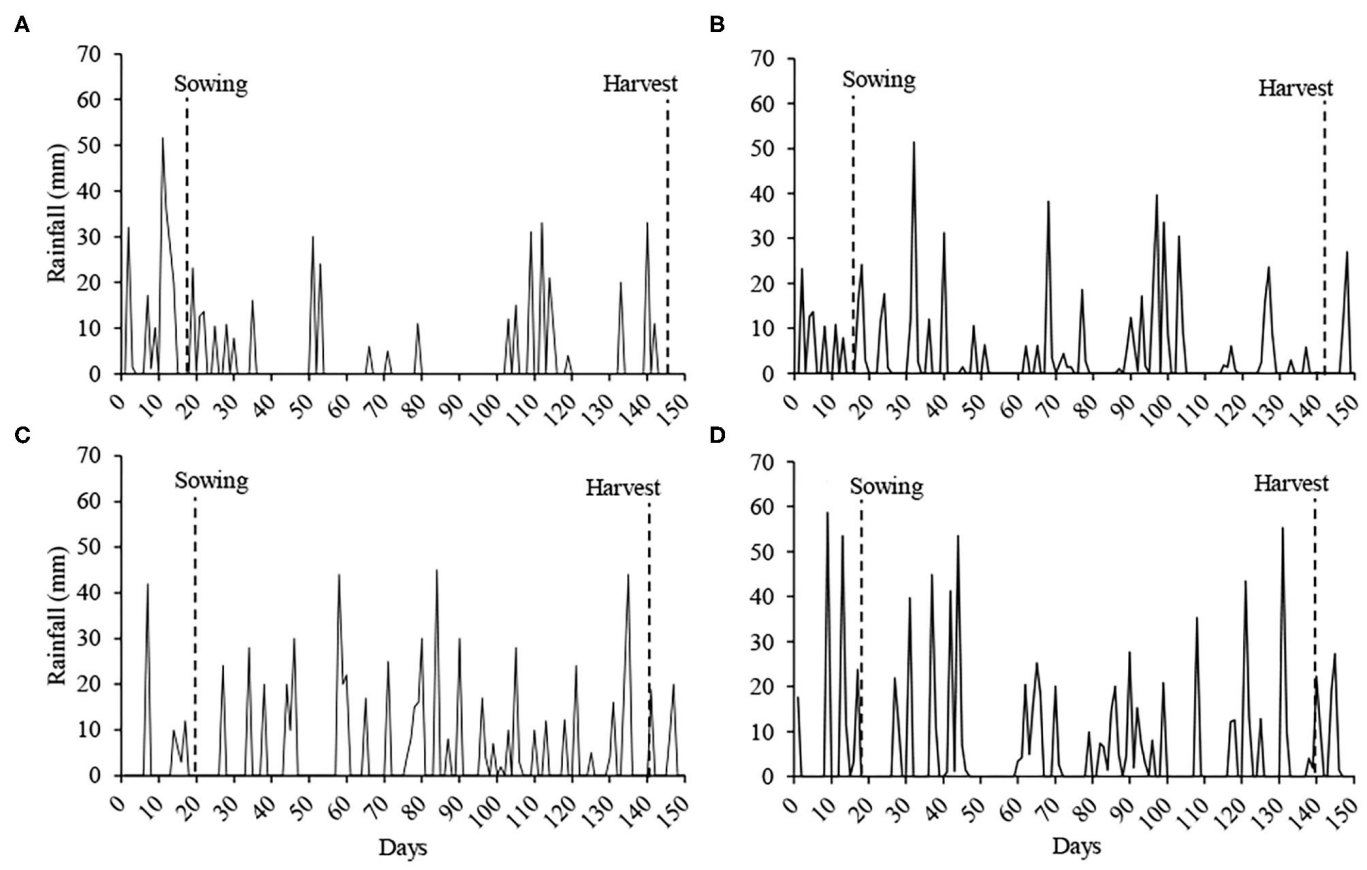
Rainfall during the soybean cycle in the experiments. **Sites 1** and **2 (A)**, **Site 3 (B)**, **Sites 4** and **5 (C)** and **Site 6 (D)**. Source: Information collected on the property of the experiments of **Sites 1**, **2**, **4**, and **5**, and Embrapa Wheat (2020) for **Sites 3** and **6**.

Rainfall during the cycle was 360.8 mm at **Sites 1** and **2**, 537.8 mm at **Site 3**, 688.2 mm at **Sites 4** and **5**, and 783.7 mm at **Site 6** (Figure 1).

### Soil Chemical Attributes

To evaluate soil chemical attributes (Table 2), the soil was collected in the sowing line up to 60 cm deep in layers of 5 cm, totaling 12 samples per trench.

**Table 2:** Methods used to determine the chemical attributes of the soil.

**Soil chemical attribute**	**Unit**	**Method of analysis**
Hydrogenionic potential (pH)	–	Water 1:1 ^1^
Phosphorus (P); Potassium (K)	mg dm^−3^	Mehlich I ^2^
Organic matter (OM)	%	Wet digestion ^3^
Calcium (Ca); Magnesium (Mg); Aluminum (Al)	cmol_c_ dm^−3^	KCl 1 mol L^−14^
Manganese (Mn); Zinc (Zn); Copper (Cu)	mg dm^−3^	KCl 1 mol L^−14^
Sulfur (S)	mg dm^−3^	CaHPO_4_ 500 mg L^−1^ of P ^5^
Boron (B)	mg dm^−3^	Hot water ^6^

1 *Jackson (1985)*.

2 *Mehlich (1953)*.

3 *Nelson and Sommers (1996)*.

4 *Bortolon and Gianello (2010)*.

5 *Hoeft et al. (1973)*.

6 *Parker and Gardner (1981)*.

The soil of this experiment contains a high iron oxide content by means of hematite that causes red color in the soils (Schaefer et al., 2008). Thus, iron analysis in soils is not common in Brazilian soils. However, it is important to remember the competition between aluminum and iron in soils, and in low soil pH conditions, aluminum toxicity may increase (Schaefer et al., 2008).

### Soil Physical Attributes

Samples of deformed soil and preserved structures were collected every 5 cm depth.

The granulometric analysis was performed by the pipette method (Embrapa, 1997). The volumetric cylinders for soil collection with the preserved structure were 5 cm in diameter and height and by means of the quotient of the dry mass of the soil by the volume of the cylinder, the soil density (SD) was determined (Embrapa, 1997).

The relative density of the soil consists of the division of the SD by the maximum soil density (SDmax). The SDmax was determined as a function of clay content (Marcolin and Klein, 2011).

To determine soil moisture in the field capacity (FC), the cylinders with saturated soil were arranged in Haines funnels at the potential of 6 kPa (60 cm). After the humidity was constant, the cylinders were weighed and brought to the air circulation oven at 105°C. Thus, it was possible to determine the soil gravimetric moisture and when multiplied by the SD, volumetric moisture was obtained (Klein, 2014). The permanent wilting point (PWP) was determined by an equation that considers the clay content of the soil (Klein et al., 2010).

Total porosity (TP) was determined by the equation proposed by Embrapa (1997). Pores were classified into macropores (>0.05 mm), micropores (0.05–0.0002 mm), and cryptopores (<0.0002 mm) and determined by increasing stresses in porous plate funnels. In the 6 kPa stress, the macropores (Embrapa, 1997) and cryptopores (1,500 kPa−150 m) were determined using the equation that considers the clay content of the soil (Klein et al., 2010). The micropores were determined by the difference between the stresses of 6 and 1,500 kPa.

The soil mechanical resistance to penetration was determined in the laboratory, using an electronic penetrometer (MARCONI, model MA-933) with a constant velocity of 0.17 mm s^−1^, equipped with a cell of 200 N and rod with a cone of 4 mm base diameter and semi-angle of 30°, and the data collected every second of penetration.

### Soil Biological Attributes

Microbial biomass and basal soil respiration were evaluated using the colorimetry methodology developed by Bartlett and Ross (1988). Microbial biomass analysis consists of fumigated and non-fumigated samples in the chloroform presence. For basal respiration, the soil was incubated in glass flasks sealed to absorb CO_2_ that the soil released. The metabolic quotient was obtained by the relationship between basal respiration and microbial biomass carbon (Anderson and Domsch, 1993).

### Root Growth

The roots were collected up to 45 cm depth, in 5 cm layers, totaling nine samples per trench. From 45 cm depth, the presence of the root was practically non-existent.

The roots were collected with an iron structure that presented dimensions of 45 × 9.25 × 5 cm in length, width, and depth, respectively. These dimensions were determined according to the spacing between rows (45 cm) and the plant density of 240,000 plants, which represents the spacing between plants in the line of 9.25 cm. So, the volume of soil that theoretically represents the sampling of a plant was collected. The roots were collected up to 45 cm deep in 5 cm layers, totaling nine samples per trench. Desiccation was performed before soybean sowing, there were no weeds in the experiments. Only live roots were collected, that is, roots of the predecessor soybean crop, and possible roots of the soybean itself that were killed were discarded.

The trenches were made transversal to the sowing line so that the sowing line was the center of the sample (45 cm). Soil separated from the roots was done by washing with running water. A 0.7 mm mesh sieve was used so that there was no loss of very thin roots and tweezers were used to remove all roots from the sieve. After this procedure, the roots were analyzed with the WinRhizo Software® determining the length (m), volume (m^3^ ha^−1^), thin root (m), medium (m), and thick (m), surface area (cm^2^), and root diameter (mm). The classification of the thin, medium, and thick root was 0–0.5, 0.5–2, and 2–4.5 mm, respectively, and represent how much of the total length of the roots presented these diameters. These roots were dried at 65°C for dry mass determination (kg ha^−1^). The data of root volume and dry mass were transformed to the hectare, considering that the volume collected from the soil with roots was 0.00208 m^3^.

### Statistical Analysis

The mean was performed for chemical, physical, and biological attributes and for root growth, and presented in graphical form for each soil depth evaluated. It was carried by Principal Component Analyses (PCA) to explain the data variation and determine which soil attributes interfere in root growth. The radicial growth variables were considered supplementary variables to verify their behavior in relation to the others, without these being part of the initial analysis of PCA (Graffelman and Aluja-Banet, 2003; Vicini et al., 2018). Their supplementary use posterior to the analysis can, however, be highly informative. In ecological community studies one often analyses abundance data and tries to relate ordinations to environmental data in a second step (Graffelman and Aluja-Banet, 2003), what in ecology studies is known as indirect gradient analysis (Braak and Prentice, 1988). Thus, we performed a weighted PCA of environmental variables and represented the root growth variables as supplementary. Since root growth is a response of the chemical, physic, and biological attributes of the soil.

## Results

### Soil Chemical Attributes

**Figure 2:**
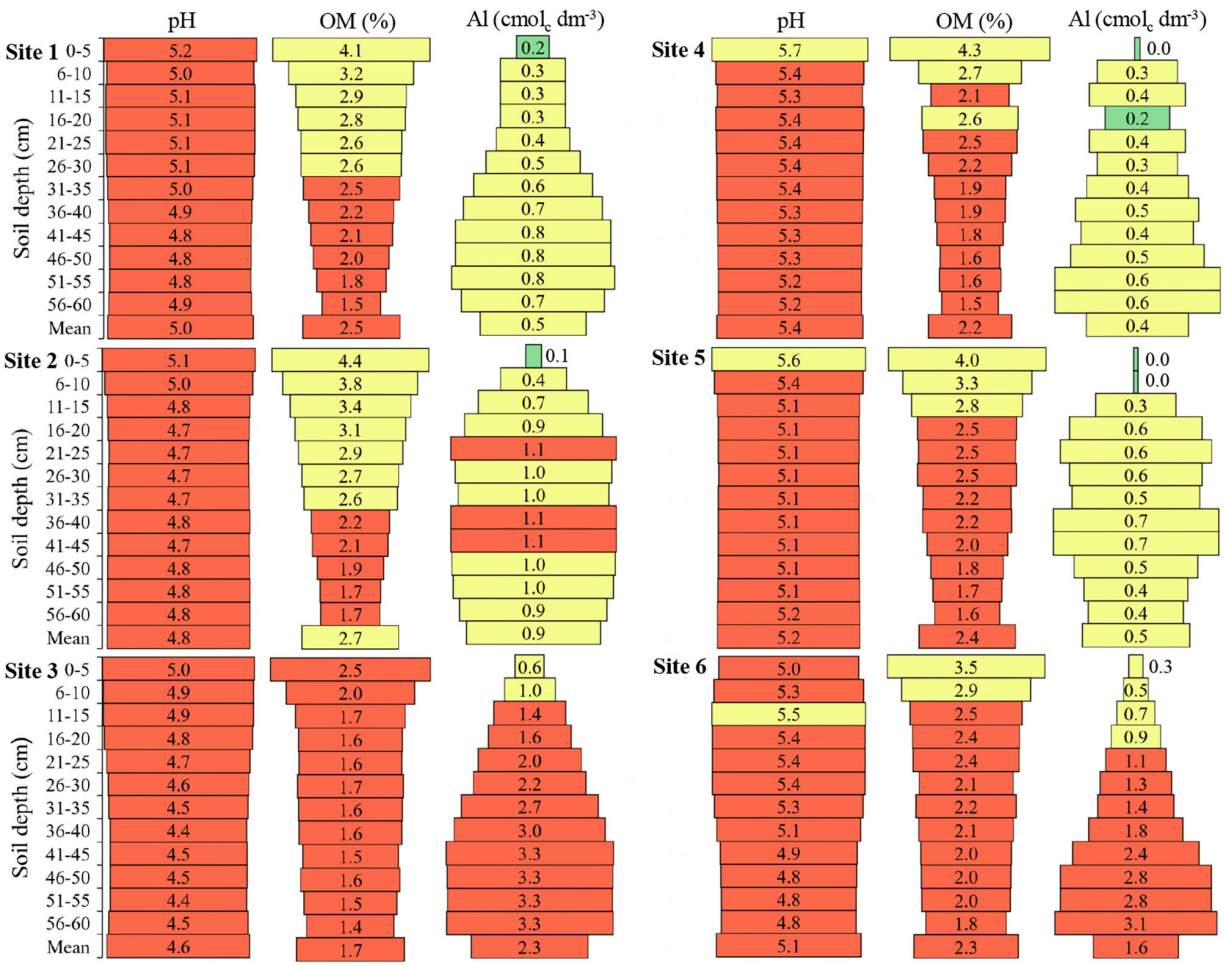
Hydrogenionic potential (pH), organic matter (OM), and aluminum (Al) in the six sites and at different soil depths. Green color: adequate values (pH 6.0–6.5; OM ≥ 6.0; Al ≤ 0.2); Yellow color: intermediate values (pH 5.5–5.9; OM 2.5–6.0; Al 0.3–1.0); Red color: inadequate values (pH ≤ 5.4; OM ≤ 2.4; Al ≥ 1.1) to soils of South of Brazil (CQFS, 2016).

The maximum pH variation between depths was 0.7 at **Site 6**, with a mean of 5.1 (Figure 2). The pH in the first layers of the soil was higher when compared to deeper layers, in general, it was observed that up to 30 cm depth, values above the mean are found; however, in all sites and depths were observed pH values below the adequate for soybean crop (pH of 6.0).

The OM, in all places, presented a greater amount of 0 to 5 cm in relation to the other depths, up to 30 cm depth the amount of OM was equal to or above the mean of each site (Figure 2). **Sites 3** and **6** presented the lowest OM values in the layer from 0 to 5 cm and **Site 3** presented the lowest mean OM among all sites; consequently, these sites presented the smallest OM difference in the soil profile. From the colors, it is possible to observe that no site and no depth evaluated presented high OM content, and **Site 3** only presented low OM values.

Al has the opposite behavior to OM in the soil profile, presenting the highest values in the deepest layers (Figure 2). **Sites 3** and **6** presented the highest Al means among the sites, with high values from 15 cm depth. Only **Sites 1, 4**, and **5** did not present inadequate Al values.

**Figure 3:**
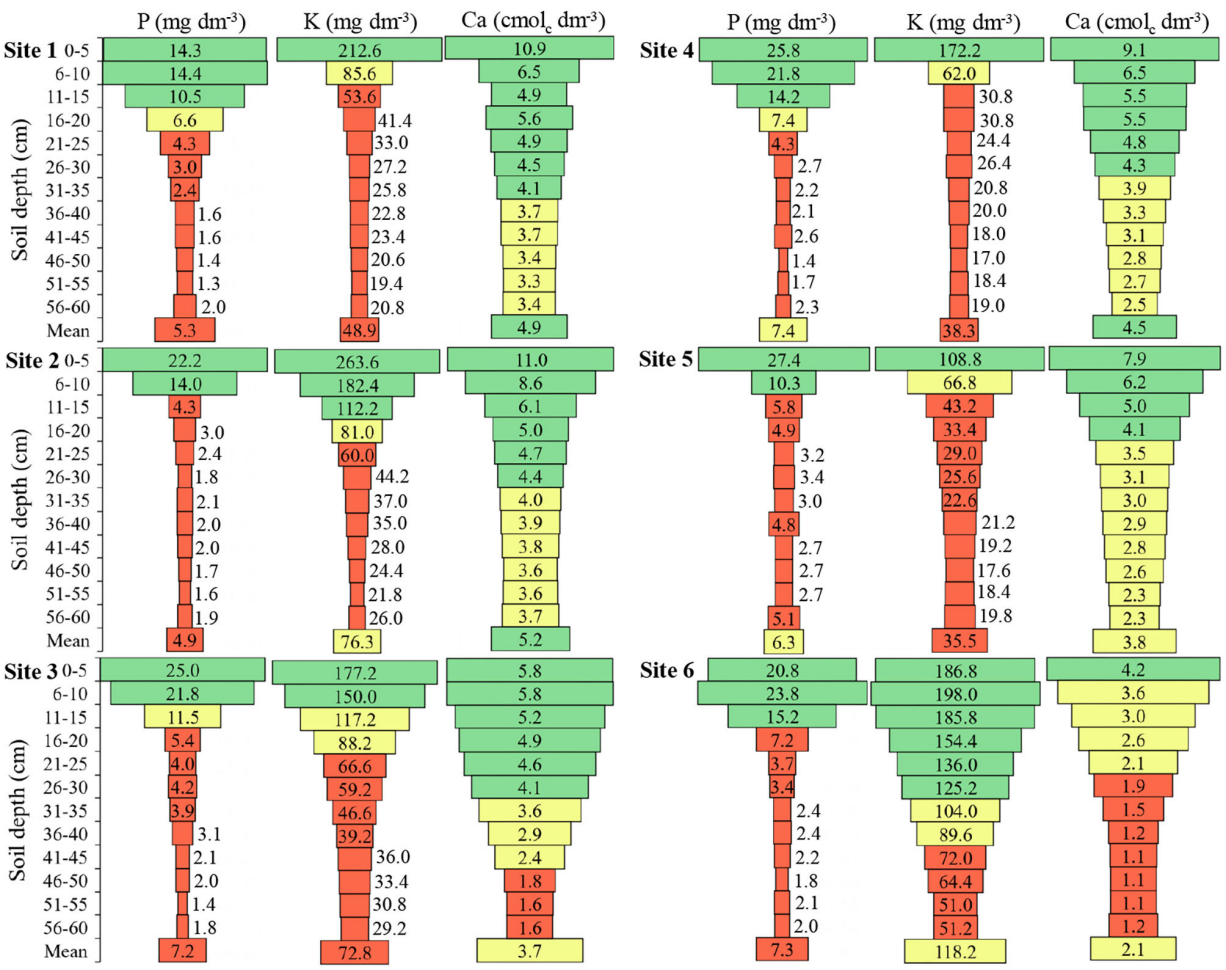
Phosphorus (P), potassium (K), and calcium (Ca) in the six sites and at different soil depths. To **Sites 1, 2, 4**, and **5**: Green color: adequate values (P ≥ 9.1; K ≥ 91.0); Yellow color: intermediate values (P 6.1–9.0; K 61–90); Red color: inadequate values (P ≤ 6.0; K ≤ 60). To **Sites 3** and **6**: Green color: adequate values (P ≥ 12.1; K ≥ 121); Yellow color: intermediate values (P 8.1–12.0; K 81–120); Red color: inadequate values (P ≤ 8.0; K ≤ 80) to soils of South of Brazil (CQFS, 2016).

The P, K, and Ca presented similar patterns in the soil profile, with higher concentrations in the soil surface layers (Figure 3). The P amount in the 0 to 5 cm depth was 2.2, 7.4, 4.6, 3.5, 5.6, and 2.9 times higher in relation to 16 to 20 cm depth, in **Sites 1, 2, 3, 4, 5**, and **6**, respectively. **Sites 1, 4**, and **6** presented high *P*-values up to 15 cm depth, while **Sites 2, 3**, and **5** up to 10 cm depth, and below these depths, the P concentration in the soils was insufficient for the soybean crop.

The K presented in 0 to 5 cm depth was 5.1, 3.2, 2.0, 5.6, 3.3, and 1.2 times higher in relation to 16 to 20 cm depth, in **Sites 1, 2, 3, 4, 5**, and **6**, respectively (Figure 3). With similar behavior of the P in the soil, the concentration of K was high in the first centimeters of depth and becomes low with increasing depth.

The Ca presented in the 0 to 5 cm depth was 1.6, 1.9, 1.6, 1.7, 2.2, and 1.2 times higher in relation to 16 to 20 cm depth, at **Sites 1, 2, 3, 4, 5**, and **6**, respectively (Figure 3). **Sites 3** and **6** presented low levels of Ca in depth.

**Figure 4:**
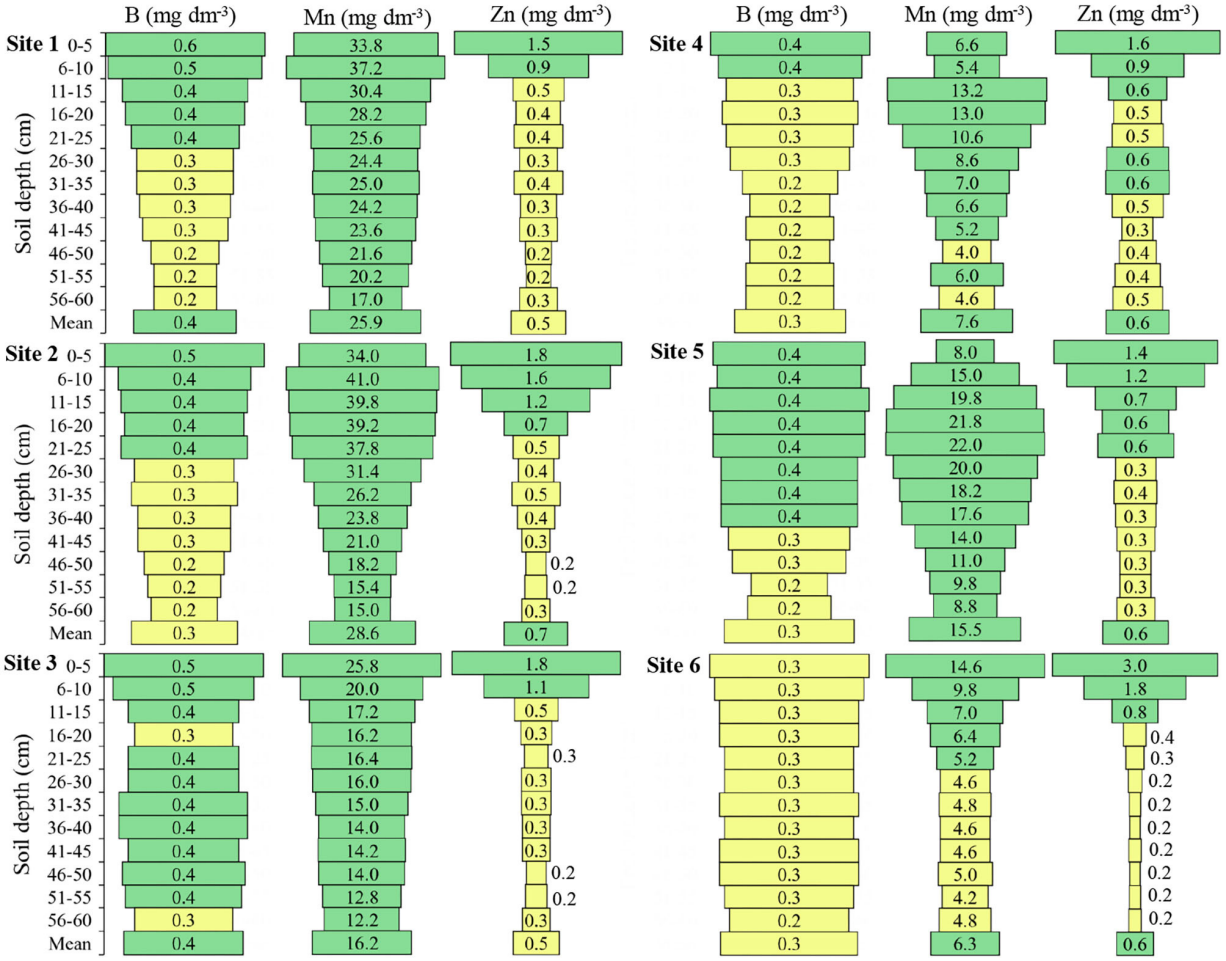
Boron (B), manganese (Mn), and Zinc (Zn) in the six locations and at different soil depths. Green color: adequate values (B ≥ 0.4; Mn ≥ 6.0; Zn ≥ 0.6) Yellow color: intermediate values (B 0.2–0.3; Mn 2.5–5.0; Zn ≥0.2–0.5); Red color: inadequate values (B ≤ 0.1; Mn ≤ 2.4; Zn ≤ 0.1) to soils of South of Brazil (CQFS, 2016).

Boron presented above-mean concentrations at **Site 1** until 25 cm, **Site 2** until 45 cm, **Site 3** until 55 cm, **Site 4** until 30 cm, **Site 5** until 50 cm, and **Site 6** until 55 cm depth (Figure 4). **Site 6** presented in all depths the mean content of B, while the other sites presented in some depths the mean content.

The mean of Mn at **Sites 1** and **2** was 25.9 and 28.6 (mg dm^−3^), respectively, at **Sites 3** and **5** was 16.2 and 15.5 (mg dm^−3^) respectively; while **Sites 4** and **6** presented the lowest means, 7.6 and 6.3 (mg dm^−3^), respectively (Figure 4). **Site 4** presented medium Mn content at depths of 46 to 50 cm and 56 to 60 cm and **Site 6** presented medium Mn content from 26 to 60 cm depth; the other sites presented high Mn content at all depths.

The Zn presented a higher concentration in the soil surface layers compared to the deep layers, and the mean concentration of Zn up to 60 cm depth varied little between the sites, with a maximum mean of 0.7 (mg dm^−3^) at **Site 2** and a minimum mean of 0.5 (mg dm^−3^) at **Sites 1** and **3** (Figure 4). In all sites, the Zn concentration at the highest depths in relation to the soil surface was intermediate for the soybean crops.

### Soil Physical Attributes

**Figure 5:**
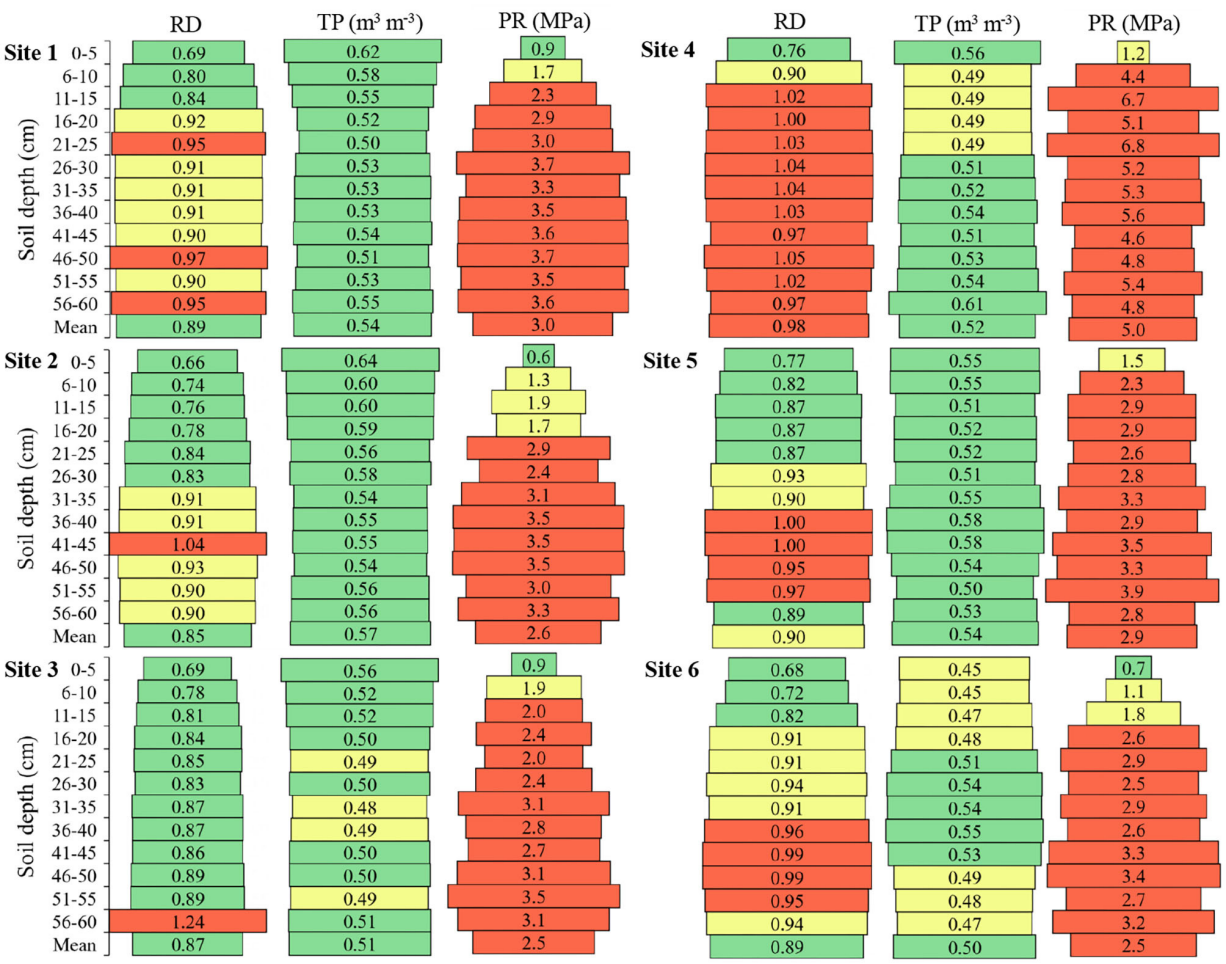
Relative density (RD), total porosity (TP), and soil penetration resistance (PR) in the six sites and at different soil depths. Green color: adequate values (RD < 0.89; TP > 0.50; PR < 1.0); Yellow color: intermediate values (RD 0.90–0.94; TP 0.45–0.5; PR 01.0–1.9); Red color: inadequate values (RD > 0.95; TP < 0.45; PR > 1.9). Adapted from Busscher et al. (2000), Reynolds et al. (2002), and Broch and Klein (2017).

There was an increase in soil relative density in the deeper layers, in relation to the surface layers (Figure 5). The highest mean of soil relative density among the sites was in **Site 4** with 0.98, then **Site 5** a with mean of 0.90, **Sites 1** and **6** a with mean of 0.89, **Site 3** a with mean of 0.87, and **Site 2** with a mean of 0.85. Noteworthy for **Site 4**, which presented soil relative density values that limit radicial growth from 6 cm deep, and to **Site 3**, which presented only a soil depth with the relative density that limits root growth.

Soil total porosity presented a lower mean at **Site 6** (0.50 m^3^ m^−3^) and higher mean at **Site 2** (0.57 m^3^ m^−3^) (Figure 5). Overall, the soils presented a pore volume close to the desired one which is 0.50 m^3^ m^−3^, and the lowest mean depths between the sites were exactly 0.50 m^3^ m^−3^.

The soil penetration resistance of **Sites 1**, **2**, **3**, **5**, and **6** presented a variation of 0.51 MPa between the highest and lowest mean found in these locations (Figure 5). **Site 4** presented a superior difference of 2.01, 2.41, 2.5, 2.1, and 2.52 MPa of **Sites 1**, **2**, **3**, **5**, and **6**, respectively. All sites showed resistance to penetration of the roots from the first centimeters of soil depth.

**Figure 6:**
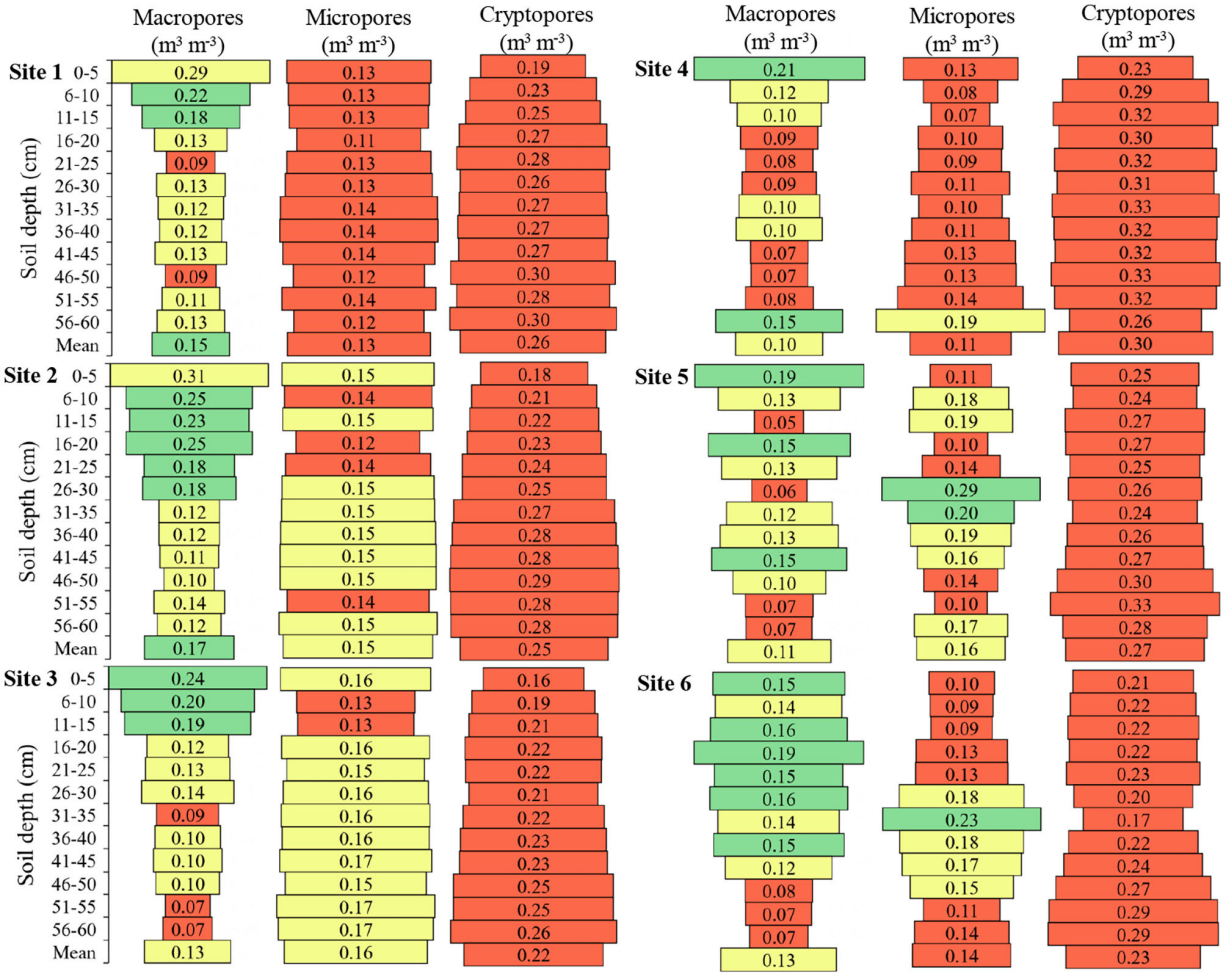
Macropores (>0.05 mm), micropores (0.05–0.0002 mm), and cryptopores (<0.0002 mm) of the soil in the six sites and at different soil depths. Green color: adequate values (Macropores 0.15–0.25; Micropores 0.20–0.30; Cryptopores ≤ 0.10); Yellow color: intermediate values (Macropores 0.10–0.14 or >0.25; Micropores 0.15–0.19 or >0.30; Cryptopores 0.10–0.15); Red color: inadequate values (Macropores <0.10; Micropores <0.15; Cryptopores >0.15). Adapted from Reynolds et al. (2002) and Reynolds et al. (2009).

Macropores were present in higher volume in the upper layers of all sites, compared to greater soil depth (Figure 6). The means were 0.15, 0.17, 0.13, 0.10, 0.11 and 0.13 m^3^ m^−3^ for **Sites 1**, **2**, **3**, **4**, **5** and **6**, respectively. **Site 2** had the highest mean macropores among the sites, being 41.2% higher than **Site 4**, 35.3% higher than **Site 5**, 23.5% higher than **Sites 3** and **6**, and 11.8% higher than **Site 1**.

The micropores presented higher uniformity of distribution between the layers in **Sites 1**, **2**, and **3**, compared to the other sites (Figure 6). At **Site 4** the largest volume of micropores was found from 56 to 60 cm deep, at **Site 5** from 26 to 30 cm deep, and at **Site 6** from 31 to 35 cm deep. The micropores means were approximately half of the values considered adequate.

The volume of cryptopores was higher at **Site 4**, in relation to the other sites and the smallest volumes of cryptopores are in the upper soil layers (Figure 6). The cryptopores values in all locations were higher than the macropores and micropores values.

### Soil Biological Attributes

**Figure 7:**
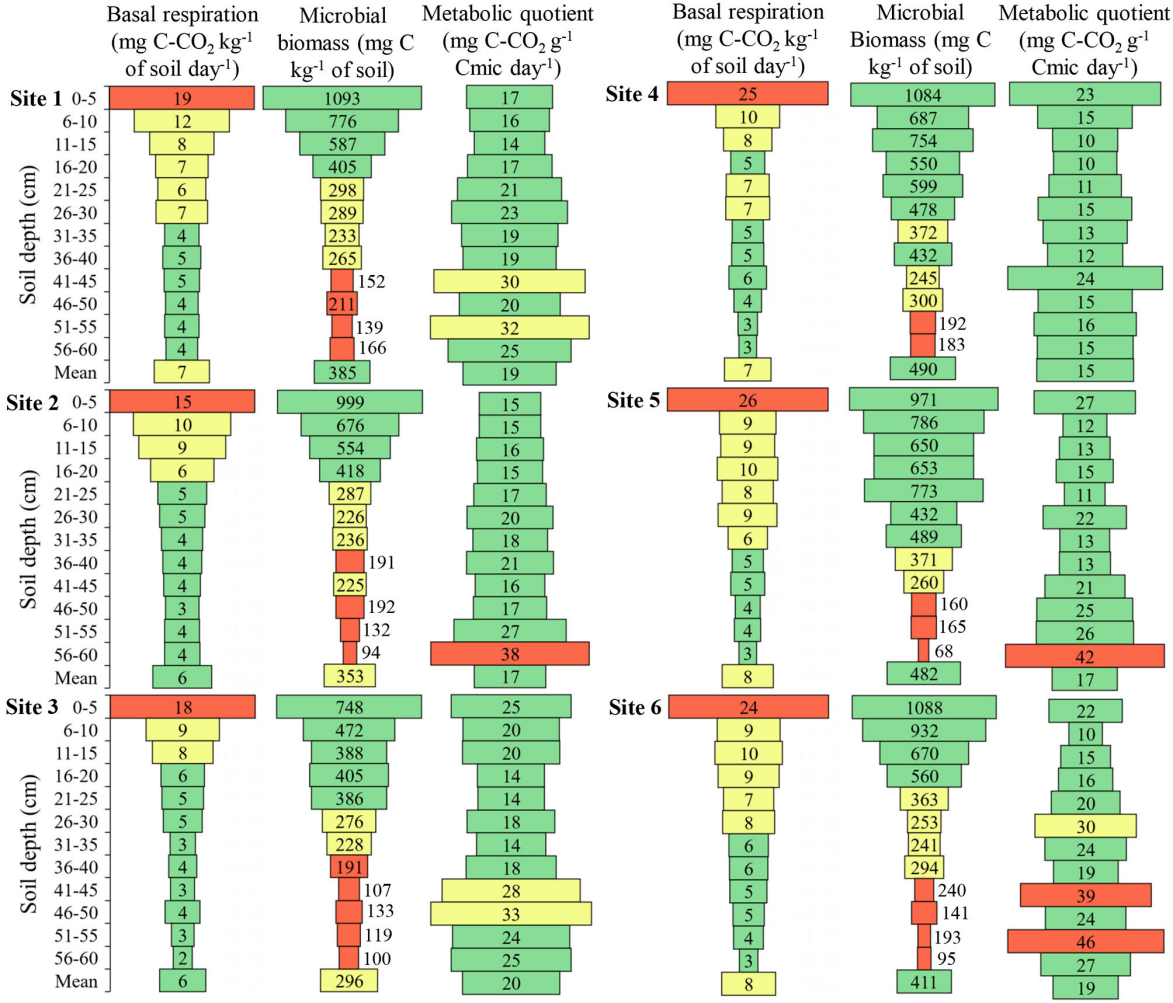
Basal respiration, microbial biomass, and metabolic quotient of the soil in the six sites and at different soil depths. Cmic, Microbial biomass carbon. Green color: adequate values (Respiration ≤ 6.0; Biomass > 375; Quotient ≤ 28.0); Yellow color: intermediate values (Respiration 6.1**–**13.0; Biomass 215.1**–**75.0; Quotient 28.1**–**35.0); Red color: inadequate values (Respiration > 13.0; Biomass ≤ 215.0; Quotient > 35.0). Adapted from Lopes et al. (2013).

Soil basal respiration showed higher values in all sites up to 5 cm depth, being 2.48, 4.56, 3.08, 3.45, 5.05, and 3.26 times higher than the mean depths for **Sites 1, 2, 3, 4, 5**, and **6**, respectively (Figure 7). When comparing basal respiration at a depth of 6 to 10 cm, the difference was 1.19, 1.54, 1.56, 1.37, 1.07, and 1.40 times higher than the mean depths for **Sites 1, 2, 3, 4, 5**, and **6**, respectively. Thus, it is observed that the amount of C-CO_2_ released near the soil surface is above the appropriate one.

The microbial biomass, at **Site 1**, showed maximum variation between the depths of 953.8 mg C kg^−1^ of soil with a mean of 384.7 mg C kg^−1^ of soil, at **Site 2** this variation was 904.8 mg C kg^−1^ of soil with a mean of 352.7 mg C kg^−1^ of soil (Figure 7). **Site 3** presented microbial biomass of 647.8 mg C kg^−1^ of soil with a mean of 296.2 mg kg^−1^ of soil, at **Site 4** it was 900.5 mg C kg^−1^ of soil with a mean of 489.6 mg kg^−1^ of soil, at **Site 5** it was 903.0 mg C kg^−1^ of soil with a mean of 481.6 mg C kg^−1^ of soil, and at **Site 6** showed maximum variation between depths of 993.0 mg C kg^−1^ of soil with a mean of 411.2 mg kg^−1^ of soil. The difference of microbial biomass between the depths was approximately twice as large as the mean depths in all sites, with the highest values near the soil surface.

The metabolic quotient at almost all depths is within the range considered adequate, however, it is perceived that there is an increase in values with the increase of soil depth (Figure 7).

### Root Growth

**Figure 8:**
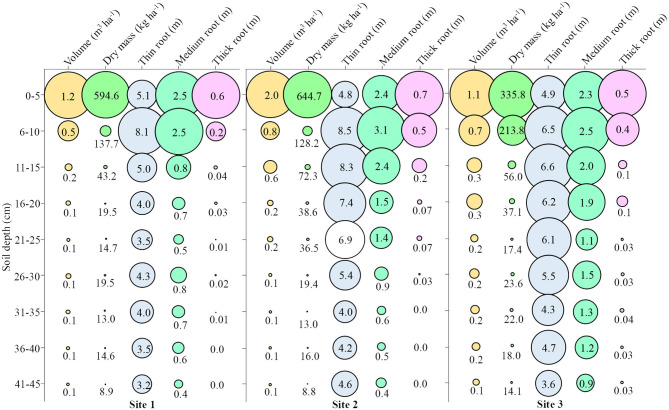
Attributes of root growth in soybean in **Site 1, Site 2**, and **Site 3** at different soil depths. Fine roots (<0.5 mm); Medium roots (0.5–2 mm); Thick roots (>2.0 mm).

At **Site 1**, the root volume up to 10 cm depth was 2.12 times greater than the sum of the other depths, which represents ~68% of the total root volume up to 10 cm depth (Figure 8 – **Site 1**). In this same line of reasoning, the dry mass presented ~85% of the total up to 10 cm depth, the thin root 32%, the medium root 53%, and the thick root 88%. The mean depth at **Site 1** was 0.28 m^3^ ha^−1^ volume, 96.19 kg ha^−1^ dry mass, 4.52 m thin root, 1.05 m medium root, and 0.10 m thick root.

At **Site 2**, ~67% of the volume was up to 10 cm depth, 79% of the dry mass, and 25% of the thin root, 42% of the middle root, and 75% of the thick root were observed (Figure 8 – **Site 2**). The mean depth at **Site 2** was 0.46 m^3^ ha^−1^ volume, 108.61 kg ha^−1^ dry mass, 6.01 m thin root, 1.47 m medium root, and 0.18 m thick root.

At **Site 3**, ~51% of the volume was up to 10 cm depth, 74% of the dry mass, and 23% of the thin root, 33% of the medium root, and 71% of the thick root were observed (Figure 8 – **Site 3**). The mean depth at **Site 3** was 0.39 m^3^ ha^−1^ volume, 81.98 kg ha^−1^ dry mass, 5.38 m thin root, 1.63 m medium root, and 0.14 m thick root.

**Figure 9:**
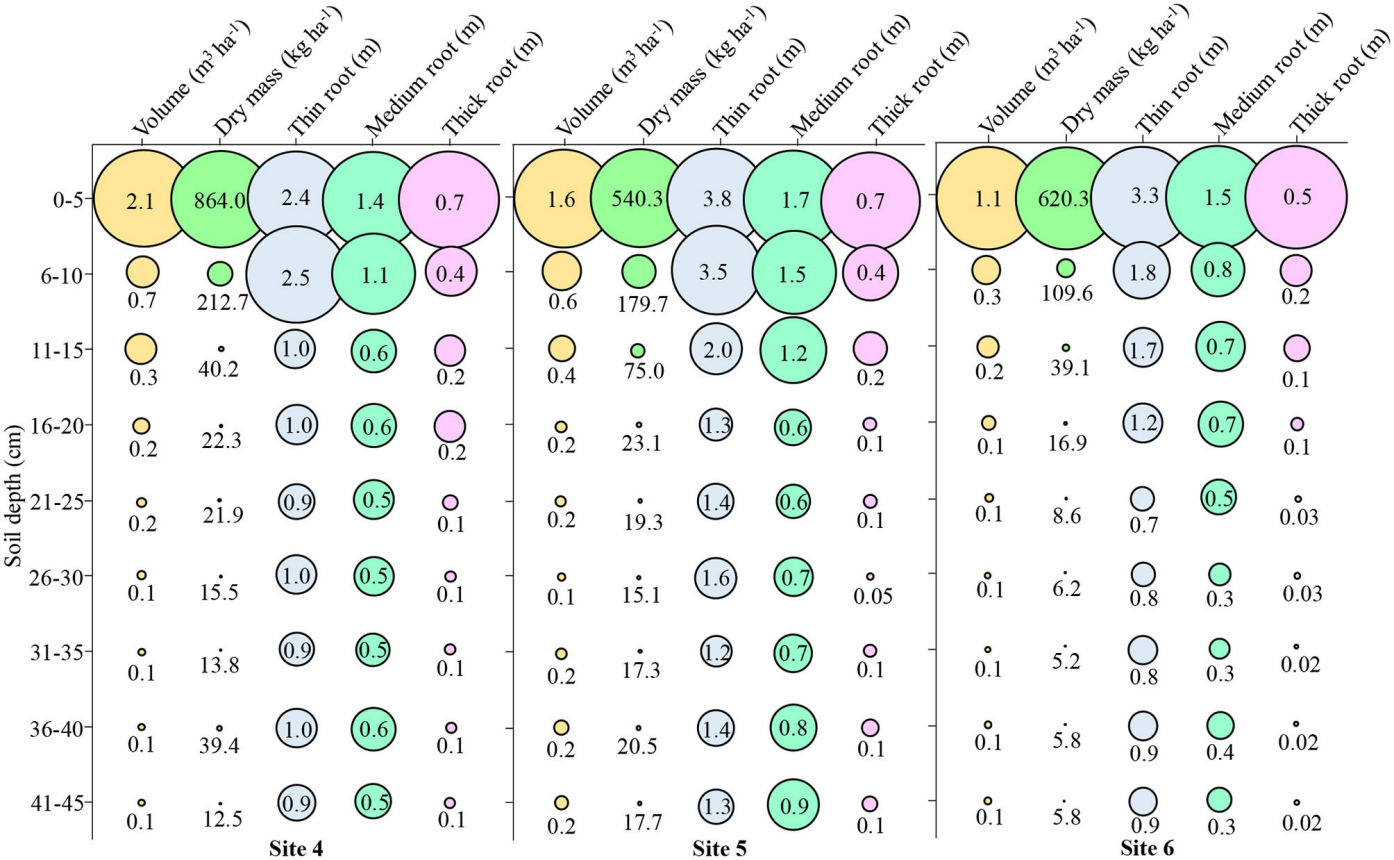
Attributes of root growth in soybean in **Site 4, Site 5**, and **Site 6** at different soil depths. Fine roots (<0.5 mm); Medium roots (0.5–2 mm); Thick roots (>2.0 mm).

At **Site 4**, ~72% of the volume was up to 10 cm depth, 88% of the dry mass and 42% of the thin root, 40% of the medium root, and 55% of the thick root were observed (Figure 9 – **Site 4**). The mean depth at **Site 4** was 0.43 m^3^ ha^−1^ volume, 135.81 kg ha^−1^ dry mass, 1.29 m thin root, 0.7 m medium root, and 0.22 m thick root.

At **Site 5**, ~59% of the volume was up to 10 cm depth, 81% of the dry mass and 41% of the thin root, 37% of the medium root, and 59% of the coarse root were observed (Figure 9 – **Site 5**). The mean depth at **Site 5** was 0.41 m^3^ ha^−1^ volume, 98.88 kg ha^−1^ dry mass, 1.94 m thin root, 0.96 m medium root, and 0.20 m thick root.

At **Site 6**, ~64% of the volume was up to 10 cm depth, 89% of the dry mass, and 42% of the thin root, 42% of the medium root and 69% of the thick root were observed (Figure 9 – **Site 6**). The mean depth at **Site 6** was 0.24 m^3^ ha^−1^ volume, 90.83 kg ha^−1^ dry mass, 1.34 m thin root, 0.61 m medium root, and 0.11 m thick root.

### Relationship of Root Growth With Soil Attributes by Principal Component Analysis

**Figure 10:**
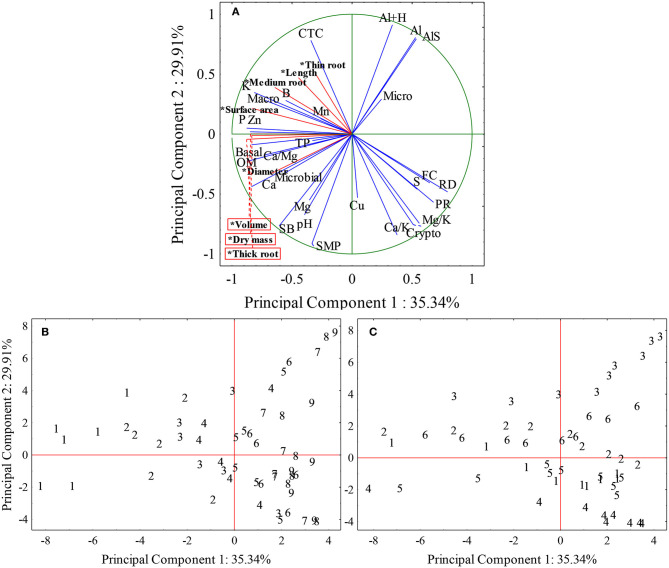
Spatial projection of vectors of chemical, physical, and biological attributes of the soil and radicial growth in different places and depths of the soil. **(A)** Cloud distribution chart of root growth variables and soil chemical, physical, and biological attributes, *Supplementary variables (radicial growth – red lines). **(B)** Depth distribution graphic. **(C)** Distribution graphic of the locations. Fine roots (<0.5 mm); Medium roots (0.5–2 mm); Thick roots (>2.0 mm), pH, Hydrogenionic potential; SMP, method of analysis and correction of soil acidity; P, Phosphorus (mg dm^−3^); K, Potassium (mg dm^−3^); OM, Organic matter (%); Al, Aluminum (cmol_c_ dm^−3^); Ca, Calcium (cmol_c_ dm^−3^); Al+H, Aluminum + Hydrogen (cmol_c_ dm^−3^); SB, Sum of bases (%); AlS, Aluminum saturation (%); Ca/Mg, Calcium, magnesium ratio; Ca/K, Calcium potassium ratio; Mg/K, Potassium magnesium ratio; S, Sulfur (mg dm^−3^); Zn, Zinc (mg dm^−3^); Cu; B; Mn; TP, Total porosity (m^3^/m^3^); Macro, Macropores (m^3^ m^−3^); Micro, Micropores (m^3^ m^−3^); Crypto, Cryptopores (m^3^ m^−3^); PR, Soil penetration resistance (MPa); FC, Field capacity (m^3^ m^−3^); RD, Relative density; Basal, Basal respiration (mg kg^−1^ of soil); Microbial, Microbial biomass (mg kg^−1^ of soil).

The variance explained by two principal components was 65.35%, when the chemical, physical, and biological attributes of the soil were analyzed together (Figure 10A). Principal component 1 is responsible for explaining 35.34% of total variance including all variables and eigenvalue of 10.25, and the variables that most contributed in factor 1 were: P, K, OM, Ca, Ca/Mg, Zn, macropores, relative density, and basal respiration. Principal component 2 presented a variance explanation of 29.91% and the variables and eigenvalue of 8.67, that most contributed to factor 2 were: SMP, Al, H+Al, CTC, sum of bases, aluminum saturation, Ca/K, Mg/K, and cryptopores.

Analyzing the correlation unit circle, variables close to each other, with a small angle between them, such as Al and AlS, FC and RD, Ca and microbial biomass, P and Zn, and K and macropores have similar representativeness in variables contribution (Figure 10A). It can also be observed that Ca, Mg, pH, SB, microbial biomass, and respiration basal showed a negative correlation with Al, as well as macropores with RD and PR. The variables close from the unit circle, more impact, and contribution they have to component 1 and 2 representing the soil analysis.

The soil depths from 1 to 4 corresponding to 0 to 20 cm depth were concentrated in the quadrants that represent the root growth, that is, they presented chemical, physical, and biological characteristics of the soil that favored the root growth of soybean plants (Figures 10B,C). All other depths showed soil attributes that disfavored the root growth.

The **Site 3** presented depths of 16 to 45 cm in the quadrant that is opposite to radicial growth with high Al content (Figures 10B,C), and the **Site 4** from 11 cm depth are in the quadrant of higher concentration of cryptopores and higher ratios of Ca/K and Mg/K, which are negatively correlated with root growth. The **Sites 1, 2, 5**, and **6** presented depths from 0 to 20 cm in the quadrants representing root growth.

## Discussion

The pH of all sites and depths presented values lower than pH 6.0, which is the critical range for the soybean crops. Furthermore, it is observed the difficulty of maintaining uniform pH in the soil profile, with lower values in the deeper layers compared to the surface layers and this occurred regardless of the organic matter and clay content in the soil. The high acidity of the soil in the subsurface layer can restrict the root growth and consequently affects the uptake of water and nutrients (Dalla Nora and Amado, 2013). One of the consequences of low soil pH is the presence of exchangeable aluminum, which reappears in soils with a pH lower than 5.5 (CQFS, 2016). There is the presence of aluminum in the six sites, mainly in **Sites 3** and **6**, which also have the lowest contents of calcium. Aluminum toxicity reduces root growth and usually, the roots are short, thick, and brittle, have few fine branches, making them inefficient in the absorption of water and nutrients from the subsoil (Rao et al., 2016; Bojórquez-Quintal et al., 2017).

The highest concentration of organic matter was in the first centimeters of soil depth; however, no depth reached a value above the critical range of 5%. The low percentage of organic matter in the soil negatively affects the root growth, because it has a direct influence on the chemical, physical, and biological pillars of the soil. Organic matter has functions in cycling and nutrient retention, forms aggregates in the soil, and is an energy source for biological activity (Gmach et al., 2020; Zhou et al., 2020).

The macronutrient phosphorus, potassium, and calcium have determining functions in the root growth of plants, such as energy, enzymatic activation, and structure of the plant wall (Fageria and Moreira, 2011). However, they are nutrients that are difficult to manage in the soil profile, presenting high concentrations in the first centimeters of depth, as well as zinc, boron, and manganese micronutrients. The distribution of phosphorus and potassium nutrients in the soil profile presented to be a problem in the six sites, because the concentration required by soybean occurred only in the first centimeters of soil depth. It can be considered that these two nutrients are at adequate levels only up to 10 cm deep. Calcium, in-depth, at **Sites 1, 2, 4**, and **5**, presented mean levels in the soil, while in **Sites 3** and **6**, calcium may have been limiting in radicial growth by low concentration, especially in **Site 6**.

The micronutrients manganese, boron, and zinc showed intermediate levels in soil depth. Manganese is an essential cofactor in the process of oxidation of water in photosystem II and the elimination of reactive oxygen species (ROS), and the deficiency of this nutrient may affect root growth due to the low availability of photoassimilates (Alejandro et al., 2020). Boron deficiency inhibits root growth by decreasing the activity of indoleacetic acid (IAA) (Li et al., 2016), and the IAA acts in the initiation and emergence of lateral roots and in the mitosis of cells (Alarcón et al., 2019). The importance of zinc micronutrient, which acts on the synthesis of tryptophan, which is a precursor required by the synthesis of IAA (Ajeesh Krishna et al., 2020) and which presented mean levels in all sites and at almost all depths.

Given the nutrients patterns in the soil, one should rethink the way of soil collection for the chemical analysis. The indication for soil collection in the consolidated no-tillage system in Brazil is from 0 to 10 cm and if there is chemical restriction to plant growth from 11 to 20 cm depth (CQFS, 2016), this type of collection may hide problems of nutrient concentration in the soil profile. When making a collection of 0 to 10 cm depth, the mean of the nutrient in the evaluated layer will be obtained and can find a value considered appropriate of the nutrient, but for example, the concentration from 5 cm depth may be less than the critical range for the full crop development. In this way, soil collection for chemical analysis should be stratified to have knowledge of the chemical composition in the soil profile and from this plan the most appropriate management for each situation. When thinking about high yields and soil profiles of this work, one notices the need to evaluate the soil in greater depth. In this way, it is held that soil collections are carried out for chemical analysis of 0 to 5 cm, 6 to 10 cm, 11 to 20 cm, and 20 to 40 cm, in which from 0 to 20 cm it is important to evaluate the general chemical condition, especially if there is a gradient of phosphorus, calcium and potassium and 20 to 40 cm, it is interesting to evaluate mainly if there is the presence of aluminum, cations, and sulfur.

The relative density of the soil was estimated because it considers the soil texture, since sandy soils present higher density than clay soils, while the density of silt soils is between sandy and clayey (Libardi, 2005). The soil density (g cm^−3^) may not match the real restriction that the soil presents to the root growth. It is noted that the densities of the soils in-depth presented restriction to the root growth, but the total porosity of the soil does not match this, because the values found of total porosity were high. The explanation for this higher relative density in depth is in the volume of macropores, micropores, and cryptopores.

The total porosity of the desired soil is close to 50%, being ~33% macropores and 66% micropores (Reynolds et al., 2002). What is observed in the six sites is that this proportion of pore size is not adequate. The volume of macropores was adequate at some depths near the soil surface. The micropores volume is almost half of the desired and the cryptopores that do not even appear in the classification of Reynolds et al. (2002), are the pores with the highest volume in the soils. It is explained why the density increased in depth even with the appropriate total pore volume. In-depth, macropores decrease and increase micropores and to a greater extent cryptopores, making the soil denser. In soils with different texture classification and management, values considered adequate for the plant-available water capacity of ≥0.15 and for macropores of ≥0.07 were reached (Reynolds et al., 2009).

The low volume of macropores can result in oxygen-deficient soil for use of soil roots and microorganisms. The desired macropore value is 0.20 m^3^ m^−3^ and considering that the soil air has a concentration of 21% oxygen, it can be considered that 4.2% of oxygen per m^3^ of soil is adequate value. The concentration of 10% oxygen in the air begins to compromise plant growth and the development of some microorganisms (Torres et al., 1993; Kuzma et al., 1999). Thus, can consider the critical limit for plant development of 2.0% oxygen per m^3^ of soil (0.20 m^3^ m^−3^ of macropores x 10% oxygen concentration). Therefore, we can consider, the lower limit of macropores for storage of oxygen sufficient to plants of ~0.10 m^3^ m^−3^, however, it should be remembered that the roots do not occupy all the space of the soil, consequently do not come into contact with all this oxygen from the soil. Therefore, in this work, we considered the value between 0.15 and 0.25 m^3^ m^−3^ of macropores adequate. Initially, it is imagined that the more macropores the better, however, the high volume of macropores limits the micropores volume and makes the soil field capacity smaller and the opposite is also true, being ideal a certain proportion in the volume of each pore size.

In general, the micropores presented lower values to desired (0.20 to 0.30 m^3^ m^−3^), limiting the water availability to plants, due to the low capacity to store it. The volume of water available to the plants, which the soils presented the capacity to store in the mean depths, ranged from 0.11 to 0.16 m^3^ m^−3^. So, the soils have the capacity to store on mean 66 to 96 mm of water available to plants, up to 60 cm deep. If we consider that the root growth occurs mainly up to 20 cm depth of the soil and the mean volume of micropores between sites up to this depth ranged from 0.095 to 0.145 m^3^ m^−3^, the capacity of the soil to store water available to plants becomes from 19 to 29 mm. The need for water increases during soybean development, reaching the maximum peak in the flowering/filling period of grains, where it needs 7 to 8 mm/day (Embrapa, 2010). Considering the volume of water available in the soil up to 60 cm deep, and the demand is 8 mm/day, the plants would remain from 8 to 12 days without water restriction, but if we consider that the roots are concentrated in the first 20 cm of soil depth, the plants would remain without water restriction for only 2 to 4 days. Therefore, in this study, we consider the value of 0.20 to 0.30 m^3^ m^−3^ of micropores to be adequate.

It was observed that the sites presented the capacity to store available water to plants (field capacity = macropores less total porosity), at the means of 37 to 42% per m^3^ of soil, but presented the capacity to store water that is unavailable to plants (permanent wilting point = cryptopores) from 22 to 30% per m^3^ of soil. Even in different sites with different managements, it was observed that the problems in pore volume are similar. In all sites, the volume of macropores and micropores are low, while the cryptopores volume is approximately the sum of macropores and micropores. Cryptopores are intra-aggregate pores that retain water with extremely high energy and are therefore unavailable to plants (Klein and Libardi, 2002).

The resistance to soil penetration presented in general an increase with the increase of soil depth, which is causally related to the pore size. In general, the PR value of 2.0 MPa has been adopted as critical to root growth (Tormena et al., 1998). In dystrophic Red Latosol that penetration resistance of 0.85 MPa resulted in a decrease of 18% in root density and a reduction in soybean yield (Beulter and Centurion, 2004). In this study, values equal to or above 2.0 MPa were considered limiting to root growth.

Soil microorganisms can be considered bioindicators of the system. The soil that presents conditions for the development and population growth of microorganisms, it means that the soil is well-structured chemically and physically and possibly the restriction to radicial growth is low. The presence of edaphic fauna and their activities in the ecosystem is influenced by soil and cultural practices, and the simplification of ecosystems and soil degradation may decrease the density and diversity of soil biota (Chen et al., 2019). The highest biomass in the first centimeters of depth in the soil profile can be due to favorable conditions, such as a higher amount of oxygen (macropores), presence of nutrients, organic matter and pH, and lower aluminum concentration and relative density of the soil. This is justified when observing that the metabolic quotient was higher in soil depth, showing a higher consumption of C-CO_2_ per unit of microbial biomass. The values considered adequate and inadequate of the respiratory quotient were calculated from the data considered adequate and inadequate of basal breathing and microbial biomass, determined by Lopes et al. (2013). The higher metabolic quotient at **Sites 3** and **6** compared to the other sites can be justified by lower total porosity, pH, organic matter, and microbial biomass and higher aluminum content in depth.

In general, soil pH, aluminum, organic matter, penetration resistance and volume of micropores, and cryptopores were not adequate in the soil profile in the six sites studied. In addition, **Sites 3** and **6** presented more toxic aluminum and less calcium than the other sites. When comparing the root growth, **Site 3** showed no significant difference with **Sites 1** and **2**, while Site 6 showed a significant difference between **Sites 4** and **5**. In the mean of the sites in the 2018/2019 harvest (**Sites 4, 5**, and **6**) the rainfall volume was higher in 286.65 mm of the 2017/2018 crop (**Sites 1, 2**, and **3**). It is noteworthy that in the year in which the volume of rainfall was lower, in the soil that presented high aluminum content (**Site 3**), soybean presented the root system near the sites with the lowest aluminum content in the soil (**Sites 1** and **2**), while in the year with the higher volume of rainfall, the soil with higher aluminum content (**Site 6**), showed lower root growth in relation to soils with lower aluminum content (**Sites 4** and **5**). In the year that there was a lower volume of rainfall, soybean presented more fine roots in depth, compared to the year of higher rainfall volume. In **Sites 4** and **5** there were 11 days without rainfall and in Site 6 were 13 days without rainfall (beginning 27 days after sowing). At **Sites 1** and **2**, there were 15 days without rainfall (beginning 18 days after sowing) and 49 days with a volume of 22 mm (beginning 30 days after sowing). At **Site 3**, 27 days were 30 mm (beginning 23 days after sowing) and 11 days with 3.8 mm (beginning 60 days after sowing). It is observed that during the vegetative phenological stages at **Sites 1, 2**, and **3**, the plants were constantly under water restriction, so even plants that grew in soil with aluminum presented radicial growth near the sites with low aluminum content. Water stress triggers the synthesis of abscisic acid in the roots, inducing the closure of stomata and reduction in photosynthesis, but also stimulates the roots growth and lateral roots formation, through cell division and stretching (Harris, 2015).

Through the data obtained, we can define some soil attributes that explain the greater variance of soils. The chemicals attribute phosphorus, potassium, organic matter, calcium, relation calcium, and magnesium and zinc; physical attributes: macropores and relative density; and, biological attribute: microbial biomass, represent around 35% of soil variance and by joining soil pH and aluminum variables and cryptopores, we will have more 30% of this variability. That is, one can study these variables to understand the behavior of the roots because they represent the greatest variance presented between the sites and soils depths.

The deeper layers were in the quadrants opposed to root growth, which in general present chemical and physical problems in the soil, while the upper depths are arranged in the quadrants that favor root growth. The sites presented two groups, being **Site 3** located in the quadrant that presents aluminum, and **Site 4** located in the quadrant that presents soil physical problems. The other sites presented the depths in all quadrants, not forming groups. Therefore, no site stood out in the root growth because all sites, at certain depths, presented restrictions on root growth.

**Site 4** presented the lowest mean of macropores and micropores and highest mean of cryptopores, relative density and penetration resistance among all sites. When **Site 4** is compared with **Site 6**, which presented chemical problems (lower pH, Ca, S, and greater Al) in relation to **Site 4**, it is observed that the root growth was lower in the soil with chemical problems in relation to the soil with physical problems. Evidencing that in the environmental conditions of the 2018/2019 crop, chemical problems in the soil prevented the root growth of soybean more than the physical problems of the soil. Perhaps this occurred because the compacted soils have fissures and the roots were able to overcome soil physical barriers, presenting roots of larger diameter. Under the environmental conditions of the 2017/2018 crop, it is observed that soil with physical restriction (**Site 1**) presented lower radicial growth in-depth, in relation to the soil with chemical problems (**Site 3**).

All sites presented favorable conditions for root growth on the soil surface. When radicial growth is concentrated in the first centimeters of soil depth, there is the problem that in small periods without rain, the water available to plants will be insufficient to its full development, resulting in loss of the productive potential of soybean. This becomes more severe when analyzing rainfall data, which is common during soybean crop development periods with water restriction. Having greater root growth in depth does not ensures that the plant will not lose productive potential in periods without rain but ensure that this loss is mitigated.

## Conclusions

Potassium, phosphorus, calcium, zinc, organic matter, macropores, and microbial biomass are the soil attributes that are highly positively correlated with soybean root growth, and the presence of aluminum and cryptopores in soil limit the radicial growth of soybean.

Stratified soil sampling is necessary for the definition of diagnosis and generation of assertive management for the soybean root growth.

## Data Availability Statement

The raw data supporting the conclusions of this article will be made available by the authors, without undue reservation.

## Author Contributions

MM and GC: conceptualization. MM, JRS, GC, ES, VK, and JSJ: methodology. AS: software. MM: writing-original draft preparation. MM, JRS, GC, ES, VK, JSC, and AS: writing-review and editing. All authors have read and agreed to the published version of the manuscript.

## Conflict of Interest

The authors declare that the research was conducted in the absence of any commercial or financial relationships that could be construed as a potential conflict of interest.

## Abbreviations

OM: organic matter
RD: relative density
TP: total porosity
PR: soil penetration resistance
SB: Sum of bases
AlS: Aluminum saturation.
